# Heart Disease Tradeoffs: The Built Environment, Air Pollution, and Activity

**DOI:** 10.1289/ehp.120-a77b

**Published:** 2012-02-01

**Authors:** Kellyn S. Betts

**Affiliations:** Kellyn S. Betts has written about environmental contaminants, hazards, and technology for solving environmental problems for publications including *EHP* and *Environmental Science & Technology* for more than a dozen years.

In many areas urban planners have begun to incorporate physical activity considerations into neighborhood design. But simply designing cities that encourage people to be more physically active may not go far enough to protect the population from heart disease, according to a study that compares the relative risks of inactivity and air pollution exposure within a large metropolitan population [*EHP* 120(2):247–253; Hankey et al.]. The new work is one of a small but growing number of studies to compare the health impacts of exercise and air pollution.

The researchers capitalized on geocoded self-report travel diaries from a state-funded 2001 survey of more than 30,000 Los Angeles residents to execute the new study. They estimated the survey respondents’ relative risk of ischemic heart disease based on cohort studies of activity level and exposure to air pollution, and used modeled and measured concentrations of fine particulate matter (PM_2.5_), ozone, and nitrogen oxides (NO_X_) to quantify individual exposures. They relied on geographic information system data to assess participants’ neighborhoods for land-use attributes demonstrated to encourage active modes of transportation: higher population density, higher intersection density (i.e., streets that are interlinked at multiple points), and a more diverse mix of land uses. People living in neighborhoods rated as having high walkability had rates of physical activity that were 50% higher than residents of low-walkability neighborhoods.

The results indicated ischemic heart disease deaths were more strongly linked with inactivity than with air pollution. However, risk differences between high- and low-walkability neighborhoods were generally comparable for air pollution and for physical inactivity, in part because high- and low-walkability neighborhoods both experienced a relatively small proportion of participants who were classified as physically active. Neighborhood patterns differed among the pollutants, with more deaths attributed to ozone exposure in low- versus high-walkability neighborhoods, and more deaths attributed to PM_2.5_ and NO_X_ exposures in high- versus low-walkability neighborhoods.

Strengths of the study include its use of real people’s rates of physical activity; many previous efforts to address the same issues have been based on hypothetical examples. Weaknesses include its cross-sectional nature and the self-report (rather than objective measurement) of physical activity. The authors conclude that efforts to design healthy neighborhoods should account for both air pollution and physical inactivity, rather than addressing each one in isolation.

